# Long-Term Effects of Individual-Focused and Team-Based Training on Health Professionals’ Intention to Have Serious Illness Conversations: A Cluster Randomised Trial

**DOI:** 10.1080/28338073.2024.2420475

**Published:** 2024-11-03

**Authors:** Dalil Asmaou Bouba, Lucas Gomes Souza, Suélène Georgina Dofara, Sabrina Guay-Bélanger, Souleymane Gadio, Diogo Mochcovitch, Jean-Sébastien Paquette, Shigeko (Seiko) Izumi, Patrick Archambault, Annette M. Totten, Louis-Paul Rivest, France Légaré

**Affiliations:** a Department of Social and Preventive Medicine, Faculty of Medicine, Université Laval, Québec, QC, Canada; b VITAM - Centre de recherche en santé durable, Centre intégré universitaire de santé et de services sociaux de la Capitale-Nationale, Québec, Canada; c Department of Family and Emergency Medicine, Université Laval, VITAM - Centre de recherche en santé durable and Canada Research Chair in Shared Decision Making and Knowledge Translation, Québec, QC, Canada; d Department of Family and Emergency Medicine, Université Laval, VITAM - Centre de recherche en santé durable, Québec, Canada; e School of Nursing, Oregon Health & Science University, Portland, OR, USA; f Centre de recherche intégrée pour un système de santé apprenant en santé et services sociaux, Centre intégré de santé et de services sociaux de Chaudière-Appalaches, Lévis QC, Canada; g Department of Family Medicine and Emergency Medicine, Faculty of Medicine, Université Laval, Québec, QC, Canada; h Department of Medical Informatics & Clinical Epidemiology, School of Medicine, Oregon Health & Science University, Portland, OR, USA; i Department of Mathematics and Statistics, Faculty of Science and Engineering, Laval University, Québec, QC, Canada

**Keywords:** Sustainability, intention, advance care planning, Serious Illness Care Program, CPD-Reaction, health professionals

## Abstract

We aimed to measure the sustainability of health professionals’ intention to have serious illness conversations with patients using the Serious Illness Conversation Guide (SICG) after individual-focused training versus team-based training. In a cluster randomised trial, we trained healthcare professionals in 40 primary care clinics and measured their intention to hold serious illness conversations immediately (T1), after 1 year (T2) and after 2 years (T3). Primary care clinics (*n* = 40) were randomly assigned to individual-focused training (comparator) or team-based training (intervention). Average age of the 373 participants was 35–44 years, 79% were women. On a scale of 1 to 7, at T1, the mean intention was 5.33 (SD 0.20) in the individual-focused group and 5.36 (SD 0.18) in the team-based group; at T2, these scores were 4.94 (SD 0.23) and 4.87 (SD 0.21) and at T3, 5.14 (SD 0.24) and 4.59 (SD 0.21), respectively. At T3, the difference in mean intention between study groups had a significant p-value of 0.01. Intention to have serious illness conversations was lower at T2 and T3 after team-based training than after individual-focused training, with a significant difference at 2 years in favour of individual-focused training. Health professionals reported not enough time during consultations for serious illness conversations as a major barrier.

**Registration number:**

ClinicalTrials.gov (ID NCT03577002) for the parent clinical trial.

## Introduction

Health professionals need to stay abreast of continuously expanding information in the health disciplines to improve their performance, both as individuals and as teams. To maintain their licences and certifications, they are required to continually update their knowledge and skills [1]. Continuing professional development (CPD) is the most common method for health professionals to remaincurrent Organisations that develop CPD seek ways to improve the effectiveness of their training programmes [2–4]. Forsetlund et al. examined the effects of several types of CPD on health professionals in a systematic review of 215 articles, the majority of which used pre-post study designs. The authors report that educational meetings (e.g. conferences and workshops) improve health professionals’ practice and, to a lesser extent, patient outcomes; they also suggest that using multiple strategies and approaches concurrently increases training effectiveness. For example, a training programme could include strategies to increase attendance at educational meetings, give feedback, establish goal-setting contracts, and focus on outcomes that are likely to be perceived as important [5]. Several descriptive studies have been conducted to understand what is likely to influence the desired behaviour changes. Research has also demonstrated that it is possible to measure the impact of training using behavioural intention, which has been validated as a proxy for behaviour change [6,7]. Intention is influenced by measurable sociocognitive factors, namely, beliefs about capabilities, beliefs about consequences, moral norm, social influences, and social/professional role [8]. Godin et al., through a systematic review of 76 studies based on social cognitive theories, developed an integrated conceptual framework for behaviour change in health professionals using intention as a measure. Most studies evaluate CPD training by measuring intention before and directly after training [1,9]. However, learners’ intentions can fluctuate over time, influenced by changes in circumstance or context. While there is some research into long-term maintenance of intended behaviour change [10], few studies evaluating CPD have followed up to find out whether health professionals sustained their intention to adopt their newly learned behaviours over time [11–16].

Advance care planning (ACP) is a process that supports patients in understanding and sharing their values, life goals, and preferences regarding future medical care during serious illness [17]. It enables patients and families to make health care decisions that correspond to their values in the context of serious illness and a limited lifespan. ACP has been shown to have positive impacts on patients and families [18–22] and more recently on health professionals as well [23]. These benefits include reducing stress, anxiety and depression in patients, surrogate decision makers, and later bereaved family members. Moreover, discussing wishes about care as illnesses progress and at the end of life can result in a better quality of life [24]. For health professionals, ACP induces a reduction in stress levels [25]. However, health professionals face many obstacles to conducting effective ACP [26]. One barrier to ACP is that many do not feel confident in their ability to lead the serious illness conversations necessary for ACP. Many health professionals also claim they do not have the time or adequate training to implement ACP in their practices [26,27]. The Serious Illness Care Program (SICP) was developed to help health professionals engage patients and care partners in ACP [28]. This programme is a comprehensive approach to ACP that includes : 1) training and implementation support, 2) identifying appropriate patients, 3) using a structured conversation guide, 4) creating workflows that allow serious illness conversations and follow-up, and 5) revisiting the conversation and revising any related decisions as patients’ conditions and preferences change [28]. Primary care professionals are well suited to holding serious illness conversations as they know their patients and can reassess them at different stages of disease progression. They can thus align ACP and decisions made about tests and treatments with the clinical condition, wishes, and values of the patient and family [29]. However, many primary care services are team-based, whereby various health professionals share the responsibilities of patient care. As the training component of the SICP originally targeted individual practitioners rather than clinical teams [29], it was not a good fit for many primary care practices and practices we approached about implementing ACP were interested in a team-based training.

We hypothesised that a team-based approach to serious illness conversation training could enhance the uptake of ACP in primary care and moreover could improve the sustainability of this behaviour change over time. We therefore sought to assess health professionals’ intention to initiate serious illness conversations with patients, and the maintenance of their intention over the next 2 years after training using an individual-focused approach compared to after training using a team-based approach.

## Method

Results are reported according to the CONSERVE 2021 statement for cluster randomised trials conducted during the COVID-19 pandemic [30].

### Study Design

The original study was a cluster randomised trial (cRT) to compare the effectiveness of two training approaches to implementing the training component of the Serious Illness Care Program (SICP) in primary care practices [31]. The trial compared an individual clinician-focused to a team-based training approach. The main primary outcomes of the original study were patient-related measures: goal concordant care and days at home; since these outcomes refer to a different population they will be published separately. Our study focused on a secondary outcome, which was the healthcare professionals’ intention to engage in serious illness conversations as measured immediately after the training, and then 1 year and 2 years later. This clinical trial was registered with ClinicalTrials.gov (NCT03577002) and the protocol has been published [31].

### Setting, Recruitment and Participants

The study was conducted in community-based primary care clinics in the US and Canada. Seven practice-based research networks (PBRNs) that are part of the Meta-LARC consortium identified primary care practices that were interested in implementing the Serious Illness Care Program. They were trained under the supervision of a joint coordinating centre at Oregon Health & Science University and Université Laval [31]. A primary care clinic was included if all the following criteria were met: 1) it estimated it could recruit at least 30 patients to engage in serious illness conversations during the study period 2) the practice was willing and able to be randomised to the individual-focused or team-based approach; 3) it had sufficient staff to participate in the team-based group, including at least one or more role/profession in addition to primary care clinicians (e.g. medical assistants, nurses, social workers, and community health workers); and 4) its staff had not been trained in any other standardised ACP programme. Health professionals from the included clinics were all invited to participate.

### Control and Intervention

Health professionals from included primary care practices who were trained using an individual-focused approach constituted the control group and those trained using the team-based approach were the intervention group. We started with the existing training developed by Ariadne Labs and created an abbreviated version for this trial; the programme was then adapted for interprofessional teams. Each course consisted of a 1.5-h online module and a 1.5-h face-to-face role-playing session. Materials used included the Serious Illness Conversation Guide (SICG). The SICG defines common subtopics for serious illness conversations and provides a structure and patient-tested language for initial and follow-up conversations [31].

The programme was developed by Ariadne Labs, a joint centre for health systems innovation at Brigham and Women’s Hospital and the Harvard T.H. Chan School of Public Health [32]. The training includes identifying patients who need a serious illness conversation, preparing them for the conversation, holding the conversation (structured according to the SICG), creating or adapting workflows to allow the conversation and follow-up conversations to take place, and documenting the conversation in the medical records [28].

### Individual-Focused Approach (Control)

The individual-focused approach assumes that serious illness conversations take place between a single primary care clinician (i.e. physicians, nurse practitioners or physician assistants) and the patient and family. In this approach, the conversation is carried out by the clinician alone.

### Team-Based Approach (Intervention)

The team-based training was developed by the research team and based on the interprofessional shared decision-making (IP-SDM) model [11,33–40]. The team-optimised approach aims to maximise the expertise and efficient use of each member’s time by distributing the various tasks among the different health professionals. The team-based training includes establishing a common approach, recognising the contributions of the different team members, continuous inter-team communication, and identifying the organisational constraints on holding serious illness conversations for each profession [31]. Each practice was allowed to use its own team model, as long as the responsibilities for the conversation were shared. More details about the development and evaluation of the adapted interprofessional team-based training are reported elsewhere [29].

### Outcomes and Measures

The main outcome of interest of the present study was health professionals’ intention to have serious illness conversations immediately, 1 and 2 years after serious illness care training using the two different approaches. The 1- and 2-year measurement points were chosen to allow health professionals enough time to apply the learning in their clinical practice [29].

Intention was assessed using CPD-Reaction, a validated 12-item tool that assesses the impact of continuous professional development activities. The tool is a 12-item self-administered questionnaire on participants’ intention (two items) and potentially associated psychosocial variables, i.e. beliefs about capabilities (three items), beliefs about consequences (two items), moral norm (two items), and social influences (three items) [6,41]. The tool is based on Godin’s integrated model [8] and its constructs correspond to three of the four learning outcome levels proposed by Kirkpatrick’s model including Level 2 outcomes, attitude and skill improvements, which relate to intention, and Level 3 outcomes, transfer of learning to practice, which relate to clinical behaviour [42]. In a second part of our enquiry, health professionals were asked to consider how their new training in serious illness conversations would affect their practice of ACP by rating their overall experience of ACP using the SICP and SICG [31] 1 and 2  years after training using a Likert scale of 1 to 7. They also rated whether they would recommend the Serious Illness Care Program to their colleagues and their confidence in their ability to engage patients in ACP using a Likert scale of 1 to 10. Barriers to integrating the knowledge gained into their practices were rated using a Likert scale from 1 to 7. For additional qualitative results, they were asked in open-ended questions to comment on how the COVID-19 pandemic had affected their engagement in ACP with patients and families.

### Sample Size

The published original study protocol provides information on the sampling [31]. Power calculations and sample size estimates were based on expected differences in the primary outcome measures (i.e. patient reports of goal-concordant care and days-at-home) and on estimates for intracluster correlations, as practices were randomised [31].

### Randomization

Primary care practices were the unit of randomisation, stratified by PBRN and practice size. They were randomised equally to the individual-focused training group or the team-based training group within each PBRN. A biostatistician who had no contact with practices or PBRNs and who remained blinded until the completion of the primary analyses used a random number generator to randomize practices. It was not possible to blind practices, patients, or other research staff. More details are published elsewhere [29].

### Data Collection and Variables

Data were collected using self-reported questionnaires immediately after the training, then at 1 year and 2 years. Sociodemographic data were collected at the end of the training. Intention to have serious illness conversations and associated psychosocial variables of intention were collected using the CPD-Reaction questionnaire. Quantitative data on perceptions of health professionals from included primary care practices about if and how they might implement ACP, as well as barriers to their practice of ACP, were also collected at 1 and 2 years, as well as qualitative data on their perception regarding the practice of ACP in the COVID-19 pandemic context. The 1- and 2-year measurement points were chosen to allow health professionals enough time to apply the learning in their clinical practice.

## Statistical Analysis

### Quantitative Analysis

All variables were analysed descriptively. Continuous variables were described using measures of dispersion (standard deviations) and central tendency (means). Categorical variables were described using absolute and relative frequencies (n, percentages). Independence, linearity,

homoscedasticity and normality postulates were tested. Intention as measured at each time point did not follow a normal distribution but was analysed as such in the linear mixed model. As reported by some authors, this model allows for non-normality [43]. The beta coefficient (β) associated with each training approach (or study group) was used to estimate the difference in mean intention between the two groups. We then performed bivariate analysis through a linear mixed model to analyse the relationship between intention and each of the sociodemographic variables. Finally, multivariate analysis was carried out using a linear mixed model including a time effect and an interaction term between study group and time variable, which allowed us to analyse the sustainability of intention. Random effects at the primary care clinic (cluster) level were specified, allowing us to reach the study objective while considering the hierarchical structure of the study. Sensitivity and subgroup analyses were performed to address potential selection bias and loss to follow-up.

The intracluster correlation coefficient (ICC) for health professionals’ intention was calculated at each time point to assess the percentage of variance attributable to practice level (cluster) in health professionals’ intention to have serious illness conversations for both study groups.

Analyses were performed using SAS (Statistical Analysis Software) 9.4.

### Qualitative Analysis

Health professionals reported how the COVID-19 pandemic has affected how they engage in advance care planning with patients and families. Their responses to the open-ended question on this topic were analysed descriptively. Data were coded using a thematic deductive approach [44,45]. Two researchers independently mapped the response data into the thematic codes.

## Results

### Characteristics of Clinical Sites and Participants

**Figure 1.**
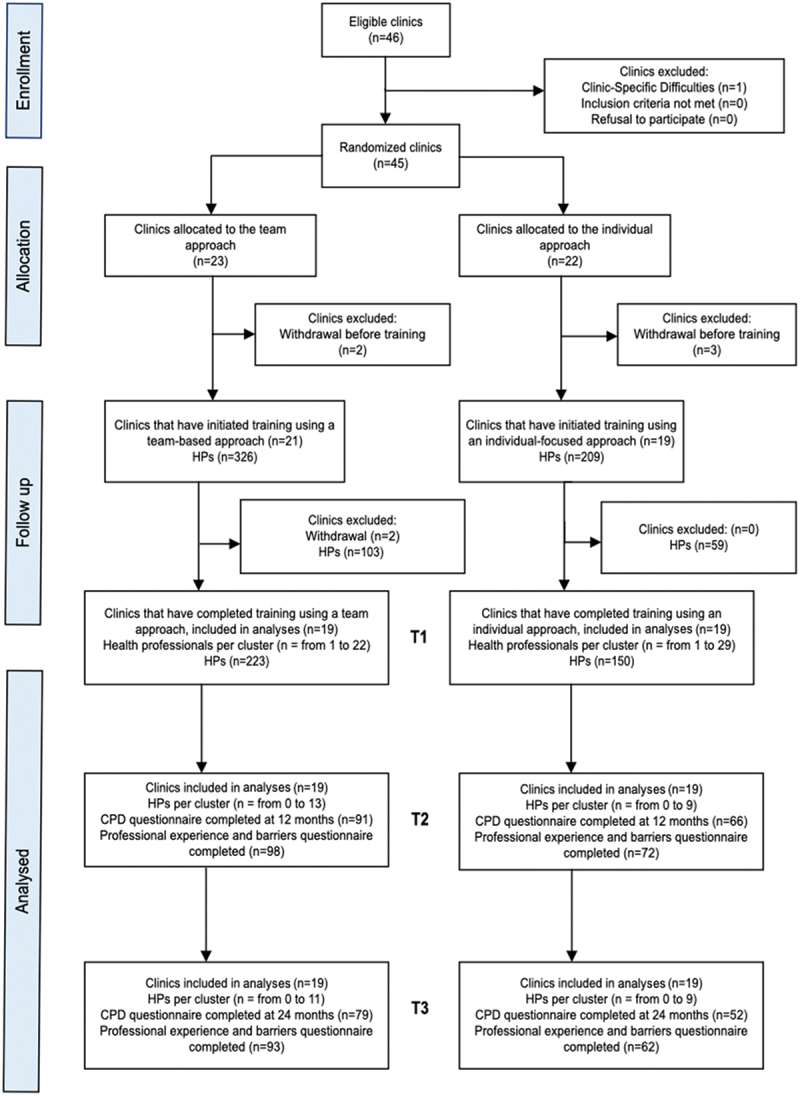
Flow chart of participants.

Forty-five (45) practices were randomised, and forty (40) practices participated by completing training (21 assigned to the team-based approach and 19 to the individual-focused approach). The characteristics of the primary care practices are summarised in Table 1 and indicate that the randomisation process at the level of the practice was successful in that the two study groups (individual-focused and team-based) were similar in key characteristics. A total of 535 health professionals were trained in serious illness conversations in the 40 primary care clinics, corresponding to a participation rate of 84.4% (38/45 practices). Of these trained health professionals, 373 completed the post-training CPD-Reaction questionnaire, for a response rate of 69.7%. One year after the training, 157 (29.3%) health professionals again completed the CPD-Reaction questionnaire, and 2 years after the training, 131 (24.4%) healthcare professionals completed the questionnaire (Figure 1). The average age of the 373 participants was between 35 and 44 years, and 79% were women. The characteristics of the participants at each time point are summarised in Table 2.

**Table 1.** Participating primary care practice characteristics.

	INDIVIDUAL		TEAM		TOTAL	
PRACTICES	N	%	N	%	N	%
Number of practices	22	100	23	100	45	100
**Country**						
United States	16	72.7	17	73.9	33	73.3
Canada	6	27.3	6	26.1	12	26.7
**Size (# of Primary Care Clinicians)**						
Small (2-5)	2	9.0	6	26.1	8	17.8
Medium (6-12)	10	45.5	9	39.1	19	42.2
Large (13-85)	10	45.5	8	34.8	18	40.0
**Geographic setting**						
Rural	8	36.4	12	52.2	20	44.4
Suburban	5	22.7	3	13.0	8	17.8
Urban	9	40.9	8	34.8	17	37.8
**Ownership**						
Hospital/health system	18	81.8	13	56.6	31	68.9
Physician or physician group	4	18.2	7	30.4	11	24.4
Federally Qualified Health Center	0	0.0	3	13.0	3	6.7
**Specialty**						
Family medicine	19	82.6	15	68.2	34	75.5
Internal medicine	3	13.0	5	22.7	8	17.8
Both Family and Internal medicine	1	4.4	2	9.1	3	6.7
**Size of Primary Care Clinicians, median** **(min to max)**	12	(3 to 40)	8	(4 to 46)	10	(3 to 46)

**Table 2.** Characteristics of the participants in the three study periods.

	T1						T2						T3					
	Study group				Total		Study group				Total		Study group				Total	
	Team		Individual				Team		Individual				Team		Individual			
	N	%	N	%	N	%	N	%	N	%	N	%	N	%	N	%	N	%
**Country**																		
USA	143	64.1	73	48.7	216	57.9	50	54.9	40	60.6	90	57.3	36	45.5	29	55.7	65	49.6
Canada	80	35.9	77	51.3	157	42.1	41	45.0	26	39.3	67	42.6	43	54.4	23	44.2	66	50.3
**Age**																		
Missing data	10	4.5	2	1.3	12	3.2	1	1.1	.	.	1	0.6	5	6.3	.	.	5	3.8
18-24	8	3.6	3	2.0	11	2.9	.	.	1	1.5	1	0.6	.	.	.	.	.	.
25-34	64	28.7	51	34.0	115	30.8	30	32.9	15	22.7	45	28.6	18	22.7	13	25.0	31	23.6
35-44	63	28.2	50	33.3	113	30.3	28	30.7	23	34.8	51	32.4	25	31.6	22	42.3	47	35.8
45-54	38	17.0	26	17.3	64	17.2	14	15.3	16	24.2	30	19.1	17	21.5	11	21.1	28	21.3
55-64	30	13.5	12	8.0	42	11.3	13	14.2	6	9.0	19	12.1	10	12.6	4	7.6	14	10.6
>65	10	4.5	2	1.3	12	3.2	5	5.4	2	3.0	7	4.4	4	5.0	1	1.9	5	3.8
Prefer not to answer	.	.	4	2.7	4	1.0	.	.	3	4.5	3	1.9	.	.	1	1.9	1	0.7
**Sex**																		
Missing data	3	1.3	.	.	3	0.8	1	1.1	.	.	1	0.6	2	2.5	.	.	2	1.5
Male	35	15.7	44	29.3	79	21.2	14	15.3	16	24.2	30	19.1	15	18.9	10	19.23	25	19.0
Female	185	83.0	106	70.7	291	78.0	76	83.5	50	75.7	126	80.2	64	81.0	42	80.7	106	80.9
**Ethnicity**																		
Missing data	6	2.7	1	0.7	7	1.9	4	4.4	1	1.5	5	3.1	2	2.5	.	.	2	1.5
Yes, Hispanic or Latino	9	4.0	4	2.7	13	3.5	4	4.4	1	1.5	5	3.1	2	2.5	2	3.8	4	3.0
No, not Hispanic or Latino	205	92.0	142	94.7	347	93.0	82	90.1	64	96.9	146	92.9	74	93.6	50	96.1	124	94.6
Prefer not to answer	3	1.3	3	2.0	6	1.6	1	1.1	.	.	1	0.6	1	1.2	.	.	1	0.7
**Race**																		
Missing data	5	2.2	1	0.7	6	1.6	3	3.3	1	1.5	4	2.5	1	1.2	1	1.9	2	1.5
White	192	86.1	128	85.3	320	85.8	85	93.4	59	89.3	144	91.7	70	88.6	47	90.3	117	89.3
Asian	13	5.8	7	4.7	20	5.4	1	1.1	.	.	1	0.6	6	7.5	1	1.9	7	5.3
American Indian or Alaska native	1	0.4	1	0.7	2	0.5	.	.	.	.	.	.	1	1.2	.	.	1	0.7
Black or African American	9	4.0	1	0.7	14	3.7	.	.	2	3.0	2	1.2	.	.	.	.	.	.
Hawaïan Native or Pacific Islander	1	0.4	.	.	1	0.3	.	.	.	.	.	.	1	1.2	1	1.9	2	1.5
Others	1	0.4	1	0.7	2	0.5	1	1.1	3	4.5	4	2.5	.	.	2	3.8	2	1.5
Prefer not to answer	1	0.4	7	4.7	8	2.1	.	.	.	.	.	.	.	.	.	.	.	.
**Education level**																		
Missing data	3	1.4	1	0.7	4	1.0	1	1.1	.	.	1	0.6	.	.	.	.	.	.
Doctoral degree	91	40.8	111	74.0	202	54.2	50	54.9	52	78.7	102	64.9	48	60.7	42	80.7	90	68.7
Master’s degree	34	15.2	31	20.7	65	17.4	9	9.8	14	21.2	23	14.6	7	8.8	10	19.2	17	12.9
Bachelor’s degree	40	17.9	.	.	40	10.7	17	18.6	.	.	17	10.8	13	16.4	.	.	13	9.9
High school	9	4.0	.	.	9	2.4	2	2.2	.	.	2	1.2	3	3.8	.	.	3	2.2
Others	46	20.6	7	4.7	53	14.2	12	13.1	.	.	12	7.6	8	10.1	.	.	8	6.1
**Profession**																		
Missing data	.	.	.	.	.	.	.	.	1	1.5	1	0.6	.	.	1	1.9	1	0.7
Primary care physician	86	38.6	101	67.3	187	50.1	45	49.4	47	71.2	92	58.6	45	56.9	37	71.1	82	62.6
Nurse practitioner	16	7.2	19	12.7	35	9.4	6	6.5	7	10.6	13	8.2	3	3.8	5	9.6	8	6.1
Nurse	54	24.2	.	.	54	14.5	19	20.8	.	.	19	12.1	16	20.2	.	.	16	12.2
Physician assistant	7	3.1	9	6.0	16	4.3	2	2.2	6	9.0	8	5.1	2	2.5	5	9.6	7	5.3
Social worker	6	2.7	.	.	6	1.6	2	2.2	.	.	2	1.2	1	1.2	.	.	1	0.7
Medical assistant	33	14.9	.	.	33	8.9	13	14.2	.	.	13	8.2	5	6.3	.	.	5	3.8
Resident	8	3.6	21	14.0	29	7.8	3	3.3	5	7.5	8	5.1	2	2.5	4	7.6	6	4.5
Others) (e.g. Pharmacists, Managers)	13	5.8	.	.	13	3.5	1	1.1	.	.	1	0.6	5	6.3	.	.	5	3.8

### Health Professionals’ Intention to Have Serious Illness Conversations

**Figure 2.**
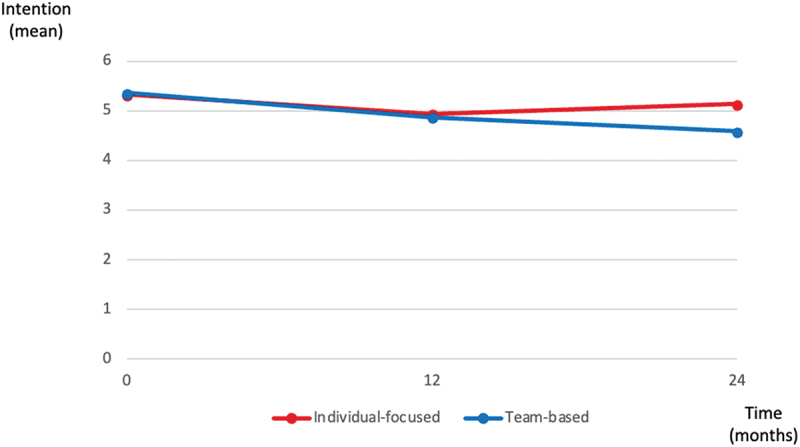
Intention trajectory according to the study group (adjusted means).

The mean intention was similar at T1 in both groups and higher in the individual-focused group at T2 and T3. On a scale of 1 to 7, the mean intention to have serious illness conversations was 6.4 (SD 0.70) for the individual-focused group, and 6.0 (SD 1.12) for the team-based group immediately after the training. It decreased to 6.0 (SD −0.88) and 5.5 (SD −1.39) for the individual-focused and team-based group, respectively, 1 year after the training (Table 3). Two years after training it increased to 6.2 (SD 0.74) in the individual-focused group and dropped to 5.2 (SD 1.53) in the team-based group (Figure 2). The difference in mean intention between the two study groups was 0.02 (CI −0.26 to 0.31), −0.07 (CI −0.49 to 0.34), and −0, 55 (−1.00 to −0.10) at T1, T2 and T3, respectively, with a p-value of 0.01 at T3 (Table 4). The p-value of the interaction term between study group and time was *p* = 0.048. Differences between the two study groups in terms of the mean of the other constructs of intention, as measured using the CPD-Reaction questionnaire, were not significant. Subgroup analyses by profession revealed that differences in mean intention between the two study groups were not significant either (data not shown).

**Table 3.** Intention and other constructs at Times 1, 2 and 3.

		T1			T2			T3		
	Study group	N	Mean	Sd	N	Mean	Sd	N	Mean	Sd
Intention	Team	223	5.3	0.1	91	4.8	0.2	79	4.5	0.2
	Individual	150	5.3	0.2	66	4.9	0.2	52	5.1	0.2
Social Influence	Team	217	4.6	1.1	92	4.6	1.1	79	4.4	1.1
	Individual	147	4.8	1.0	67	4.6	1.0	54	4.9	1.1
Beliefs about capabilities	Team	223	5.4	0.8	92	5.4	1.1	79	5.2	1.2
	Individual	150	5.6	0.8	67	5.7	0.7	54	6.0	0.6
Moral norm	Team	220	6.7	0.5	92	6.4	0.9	78	6.4	0.9
	Individual	149	6.8	0.3	65	6.7	0.5	53	6.6	0.4
Beliefs about consequences	Team	221	6.3	0.9	92	6.0	1.1	78	5.9	1.3
	Individual	149	6.5	0.6	63	6.4	0.6	54	6.5	0.6

*Sd = standard deviation.

**Table 4.** Differences in mean intention at each time point.

	Intention (difference in means)						
	Unadjusted model (N = 373)			Adjusted model* (N = 353)			
	Estimate	CI 95%	p value	Estimate		CI 95%	p value
T1	− 0.42	− 0.67; − 0.16	***0.001***	0.026		− 0.26; 0.31	0.86
	**(N = 157)**			**(N = 151)**			
T2	− 0.51	− 0.96; − 0.07	***0.02***	− 0.07		− 0.49; 0.34	0.72
	**(N = 131)**			**(N = 123)**			
T3	− 0.96	− 1.46; − 0.46	***0.0002***	− 0.55	− 1.004; − 0.10		***0.01***

*Adjusted for profession, education and ethnicity, study group-time interaction, *CI: confidence interval.

### Health professionals’ Perceptions About ACP Implementation and Barriers

In both study groups, after 1 year, health professionals felt confident in their ability to engage seriously ill patients in ACP (mean of 7.2 in the team-based group and 7.8 in the individual-based group at 1 year, and 7.3 and 8.5, respectively, for the same groups at 2 years, on a scale of 1 to 10 (1 = not at all confident, 10 = extremely confident). This trend was higher in the individual-focused group, and these results were similar after 2 years.

Health professionals reported insufficient time during scheduled appointments to address the topic of ACP, with a mean of 4.7 and 5.4, respectively, for team-based and individual-based groups at 1 year, and 4.7 and 4.6, respectively, for the same groups at 2 years (on a scale of 1 to 7: 1 = not at all important, 7 = extremely important). All of them also reported difficulty in defining the right time to engage patients in serious illness conversations, and difficulties in dealing with the uncertainty of prognosis for patients with chronic illness. These were the most important barriers to implementing ACP 1 and 2 years after training (Table 5).

**Table 5.** Perceptions and barriers, health professionals at 1 and 2 years.

PERCEPTIONS OF HEALTH PROFESSIONALS	Study group − 1 year						Study group − 2 years					
	Team			Individual			Team			Individual		
	N	Mean	Sd*	N	Mean	Sd	N	Mean	Sd	N	Mean	Sd
How likely are you to recommend the Serious Illness Care Program and advance care planning to colleagues and other primary care practices? (On a scale of 1 to 10, 1= not at all, 10= extremely)	98	7.34	2.32	72	7.78	2.04	93	7.53	2.30	62	8.27	1.60
How confident are you in your ability to engage seriously ill patients in advance care planning? (On a scale of 1 to 10, 1= not at all, 10= extremely)	97	7.21	1.97	72	7.85	1.69	93	7.30	2.02	62	8.56	1.33
Do you ask patients what is important to them? (On a scale of 1 to 7, 1= never, 7= all the time)	98	5.00	1.71	72	5.24	1.12	93	4.90	1.64	62	5.73	1.05
Do you document specific patient goals and preferences in patient records? (On a scale of 1 to 7, 1= never, 7= all the time)	98	4.72	1.92	72	5.03	1.39	93	4.70	1.93	62	5.53	1.36
Discuss next steps or plans related to ACP, for example naming a proxy, talking to family, sharing goals with other health care providers. (On a scale of 1 to 7, 1= never, 7= all the time)	98	4.43	1.67	72	5.01	1.29	93	4.48	1.56	62	5.47	0.92
Do you initiate discussions about Advance Care Planning? (On a scale of 1 to 7, 1= never, 7= all the time)	98	4.36	1.57	72	4.79	1.23	92	4.23	1.68	62	5.27	1.05
Do you talk about the future, such as how their condition may change, when things may become more difficult, or how long they may live? (On a scale of 1 to 7, 1= never, 7= all the time)	98	4.31	1.53	71	4.85	1.06	93	4.50	1.60	62	5.34	1.12
Do you have follow-up serious illness care discussions with patients with whom you initiated a conversation in the past? (On a scale of 1 to 7, 1= never, 7= all the time)	98	3.94	1.74	72	4.42	1.48	93	4.11	1.80	62	5.97	1.35
**BARRIERS** (On a scale of 1 to 7, 1= not at all important, 7= extremely important)												
Insufficient time during scheduled appointments to deal with this topic	97	4.73	1.85	72	5.40	1.65	91	4.78	1.63	61	4.64	1.78
My difficulties in dealing with uncertainty of prognosis for patients with chronic illness	97	3.67	1.43	72	3.31	1.33	91	3.51	1.43	62	3.24	1.05
My difficulties with defining the right moment to engage patients in Serious Illness Conversations	97	3.57	1.35	72	3.67	1.28	91	3.56	1.28	62	3.13	1.15
Patients not understanding or misinterpreting my reasons for bringing up the topic	97	3.44	1.29	72	3.50	1.45	91	3.53	1.42	62	3.35	1.22
Limited capacity to honour patients’ expectations for care that arise from Serious Illness Conversations	97	2.82	1.28	72	2.88	1.27	91	3.00	1.42	62	2.47	1.10
My having to deal with the emotional impact of Serious Illness Conversations on patients	97	2.80	1.23	72	2.42	1.17	90	2.92	1.17	62	2.53	1.07

*Sd: standard deviation.

### Percentage of Variance Attributable to Practice Level (Cluster) in Health Professionals’ Intention

The overall ICC was 0.18 after training, 0.34 one year after training, and 0.31 two years after training.

In the individual-focused group, ICC was, respectively, 0.09, 0.34 and 0.21 at T1, T2 and T3. In the team-based group, ICC was, respectively, 0.18, 0.34 and 0.28.

### How the COVID-19 Pandemic Affected How Health Professionals Engage in ACP with Patients and Families

During the COVID-19 pandemic, health professionals reported that patients had fewer appointments and therefore fewer opportunities to engage in ACP. However, they felt that for patients who had appointments, serious illness conversations were even more important than before the pandemic, because these patients with serious illnesses were the most vulnerable to Covid-19. This added vulnerability made it easier for health professionals to initiate the conversations. Health professionals also reported telemedicine as a challenge for initiating serious illness conversations during the pandemic, mentioning that virtual or remote communications prevented them from showing the physical signs of listening and empathy (Table 5).

Health professionals in the individual-focused group reported that it was difficult to talk with families in a meaningful way when they were not able to be physically present.

Mostly in the team-based group, participants reported not having any opportunity to engage in these discussions since the start of the COVID-19 pandemic, as they did not have time allotted in their schedule (Table 6).

**Table 6.** How the COVID-19 pandemic affected how health professionals engage in advance care planning with patients and families.

How the COVID-19 pandemic affected how health professionals engage in ACP				
Code	Illustrative quotations	2021		
		Team	Individual	Total
No patients due to Covid	“Many of the most ill patients avoided coming to the clinic to minimise their risk of exposure”.	12	10	22
More difficult with telemedicine	“Virtual/remote formats have made this more difficult. Not able to give physical cues of listening, empathy as well. Also, patients who were hard of hearing”	10	9	19
Covid emergency	“I have not had any opportunities to engage in these discussions since the start of the pandemic. We do not have time/opportunities allotted in our schedule”.	13	4	17
Workflow & priorities	“Covid has affected a lot how the whole practice engages. Our primary focus for the past year was first making everything safe here in the practice to see patients”.	12	4	16
Physical absence	“Difficult to talk with families in a meaningful way when they are not able to be physically present”.	5	10	15
No change	“It did not change my practice otherwise”.	7	7	14
Easier to start conversations in Covid context	“Easier to talk about considering the risk of complications from Covid. Discussion around Covid care sometimes opens the door to discussion elements”.	9	5	14
Easier thanks to telemedicine	“I find telephone easier – I can take better notes and may cover more thoroughly”.	5	3	8
Less discussion	“Very few visits, limited opportunities for ad-hoc ACP. Much less ACP”.	5	3	8
Negatively overall	“Negatively”	2	1	3
Unable to code		4	4	8

## Discussion

We compared the sustainability of health professionals’ intention to have serious illness conversations with their patients after individual-focused versus team-based training in serious illness conversations immediately after training and 1 and 2 years later. We found that the mean intention to have serious illness conversations was higher in the individual-focused group than in the team-based group after one and 2 years, and this difference in means was statistically significant 2 years after the training. The p-value of the interaction term between time point and study group was close to the significance threshold. In both groups, health professionals felt largely confident in their ability to engage seriously ill patients in ACP. However, they reported that not having enough time during scheduled appointments to address the topic was the main difficulty in implementing ACP 1 and 2 years after training. These results lead us to make the following observations.

First, intention in the team-based group decreased after one year and then more so after two years. In the individual-focused group, intention had decreased at year 1 but then increased in year 2. The statistically significant difference in intention in favour of the individual-focused group observed only 2 years after training suggests that an individual-focused approach could have a longer-term impact on the intention of health professionals than a team-based approach. The decline in intention to have serious illness conversations in both groups, but mostly in the team-based group at year 1, could be explained by multiple factors. The outcome was measured during the COVID-19 pandemic, when emergencies related to the pandemic may have relegated ACP to the background [46]. The impact of COVID-19 on health professional burnout has been reported [47]. Also, during the COVID-19 pandemic, health professionals from both groups reported that patients were increasingly absent from ACP-related appointments for fear of becoming infected, or simply to comply with the restrictions put in place by the government to limit the spread of the virus. Telemedicine became more frequent, which made team work harder [48,49]. If the literature reports an increase in the remote practice of medicine since Covid-19, opinions on its practice are mixed. The difficulties encountered by the health professionals in our study were also found in a study conducted before the pandemic: nurses working in telehealth in Brazil reported that technology has facilitated their professional practice, but they found it harder to communicate by telehealth, mainly due to difficulty in perceiving nonverbal signals [50]. Health professionals also mentioned they were not able to give the physical cues that signal listening and empathy. The literature reports on the importance of online ACP resources that supported community-based ACP conversations during the COVID-19 pandemic [51]. However, in a study on conducting ACP through telehealth, resident physicians hypothesised that although patients might be more relaxed for the ACP discussion in their own homes, they found it easier to convey compassion and to interpret the patient’s reactions in person [52]. It is thus possible that this shift to telemedicine undermined health professionals’ motivation for holding serious illness conversations. Some other authors report that telemedicine is in fact more useful for team-based ACP, as it is more appropriate for the tasks other than the conversation itself, such as the integration of the agreed ACP plan in medical records and online programs for preparing people for an in-person serious illness conversation, tasks which could be undertaken by various other team members [51,52]. One year later, the pandemic was under better control. The team-based approach has been widely promoted to provide effective and efficient care that integrates the skills of the different health professionals to contribute to a common goal [53–56]; and teamwork ensuring a good division of tasks and coordination has been reported by other authors as a facilitator [57,58]. However, even beyond the pandemic context, the approach presents some challenges. High levels of team turnover also may have had a negative impact on interprofessional team motivation. In the United States, a study reported a 15% turnover rate for clinicians and other staff in some primary care clinics, and an average turnover rate of 53% over a 2-year period for physician and staff turnover in Ohio primary care clinics [59]. In Canada, hospital staff turnover rates were 26% in 2021 [60]. Professional hierarchies, lack of leadership, poor inter-team communication and relationships, and lack of knowledge of other team members’ abilities and roles are factors that frequently reduce team effectiveness in patient health management. Because of these difficulties, the change to teamwork may have undermined health professionals’ confidence in engaging in serious illness conversations [61], which are complex and time-consuming. To restore this confidence, team-based training on this topic could be clearer about how the other healthcare roles can be involved in supporting ACP or tailor training to each specific role. Some of the participants in the team-based group had no experience of ACP, so the concept was new to them. This has also been described as a barrier in the literature [62]. Certain methods of training health professionals in interdisciplinary teams seem to have proven helpful, such as training focused on predefined learning objectives which address gaps in health professional training identified through in-depth literature reviews, or basing the training on discussions with expert faculty as well as on learner feedback from previous activities [63]. Also, a patient-centred format that includes a patient or patient representative is helpful to better reflect clinical practice [64]. Indeed, medical education featuring patients provides a critical perspective and brings to life real-world issues related to patient care such as advance care planning [64].

Second, the p-value of the interaction term between time point and study group was not significant, meaning that intention would not vary depending on the training approach (study group) over time. This *p* value, although very close to our significance threshold, does not allow us to formally reject the null hypothesis, especially since it is a secondary outcome with a power calculated a posteriori and less than 80%; this suggests that with higher power, we would likely have a much higher p-value which would thus be above the significance threshold, as found during sensitivity analyses [65]. This result indicates that the effect of the training approach on health professional’s intention might be constant over time and that the impact of the training approach (individual-focused or team-based) may not be different at different stages during follow-up. The effect of time on the intention would then be similar in both groups and there would likely be a main effect of time, independent of the study group, i.e. intention would change over time, but this change would be similar in both groups. The effect of the team-based training seemed to gradually diminish over time. This highlights the importance of conducting repeated measurements over an extended period to fully understand the effect of the two approaches on health professionals’ intention, although the difficulty of conducting clinical trials over long periods while maintaining high power is well known [66]. The absence of an interaction with time does not necessarily mean the absence of effect of the different training approaches over time. There may be nuances in the data that are not captured by the interaction, so further exploration of the results is necessary [65]. Analysis of repeated measures provides a more complete description of the time effect since it includes data from all time points, allowing statistical assessment of potential different response curves to the intervention over time [67]. For instance, there might be little or no improvement in the intervention group and larger improvement in the control group, as we observed in this study, or the same total improvement between the baseline and end point in both groups but faster response in one of the groups [67].

Third, although health professionals in both groups felt generally confident in their ability to engage seriously ill patients in ACP after 1 and 2 years (a trend which remained higher in the individual-focused group over time), participants in both groups reported insufficient time during scheduled appointments to address the topic of ACP as the main barrier in their practice. A study on the impacts of interprofessional training in end-of-life conversations also found that more time would help them improve their communication with patients about care objectives [68]. Teamwork, which allows for the distribution of tasks between several health professionals, should reduce the time dedicated to this task. The literature reveals that adequate training and skills transfer help health professionals in non-palliative care settings to be equipped with the appropriate attitudes, knowledge and skills for conducting ACP and to address patients’ and their carers’ needs and preferences regarding their care [69].

Lastly, the overall ICC increased over time. In each group, an increase in ICC was observed at 1 year and 2 years compared to ICC immediately after training. This reflects a higher similarity of observations within the same clusters. This could be explained by the fact that individuals within the clusters had all received the same training and had thus become more similar in their intentions. In addition, these ICC values are similar to those reported in the literature for randomised clinical trials in primary health care [70] and their scope supports the need to adjust the sample size in the future to achieve adequate power.

## Strengths and Limitations

To our knowledge, this study is the first to measure the intention of health professionals over time in the context of ACP and is among the very few to document the impact of CPD on intention after 2 years. By measuring intention over the long term, we contribute data to the ongoing debate over the intention-behaviour gap. In a similar study, self-reported behaviour matched intention measured 6 months earlier [1]. There was also no loss to follow-up of the units of randomisation, which were the clinics. Finally, another strength of this study is that our results were obtained using an adapted statistical model that considered the structure of the study and the time variable [43]. However, our study has limitations. Although healthcare professionals’ intentions to adopt a behaviour are considered a good proxy for future behaviour adoption [7], among the confounding variables were the potential fluctuation of intention over time, influenced by circumstances such as COVID-19 and professional pressures due to high staff turnover, which led to significant loss to follow-up amongclinicians and team members. However, in the second questionnaire, we also asked about behaviour itself, i.e. about how the training affected their practice of ACP 1 and 2 years after training. This was a cRT within which clusters were the units of randomisation. However, the nature of the intervention (individual-focused vs. team-based) had an impact on the presence or absence of some professions. Unlike in the control group, participants in the intervention group (team-based approach) included a significant number of nurses, medical assistants, and social workers, and a few people in other role who may not have perceived ACP and the training as directly relevent to their current role in the health care system. This may have induced a selection bias (non-compliance bias) within a cRT and not at the level of the unit of randomisation. We believe we controlled for this non-compliance bias by stratification through sensitivity analyzes y profession, which showed similar results in the two study groups. In addition, the socio-demographic profiles of the participants were not different between the groups. Finally, the reduced sample size at the level of the individual participants at 1 and 2 years after training during a pandemic resulted in a reduction of power. Strategies to reduce loss to follow-up with a larger number of participants would allow these results to be more robust and generalised.

## Conclusion

Two years after training using the team-based approach, health professionals’ intention to have serious illness conversations was no more sustainable than after training using an individual-focused approach. The complexities of the COVID-19 pandemic could have affected our results at 1 and 2 years. These results could also suggest that optimal understanding and acceptance of the role of teams in serious illness conversations, which are traditionally an individual task (i.e. one-on-one), has not yet been achieved. The shift to teamwork may also have undermined team-based health professionals’ confidence in engaging in these difficult and delicate conversations. The team-based training approach should be clear about how other team members can be involved in supporting ACP and how this could address the much-cited time barrier. Finally, for programmes to be effective, they should ensure participants can in fact put their learning into practice in their current professional roles. Indeed, it suggests future SICP training programmes could tailor the training to the specific role of each health professional in the team. The allocation of more time to ACP and ACP encounters scheduled into the clinical calendar would have the potential to both increase its sustainable implementation by health professionals and promote more engagement by caregivers and patients.

## Abbreviations

ACP: Advanced care planning
CPD-Reaction: Continuous Professional Development questionnaire
ICC: Intracluster Correlation Coefficient
PBRN: Practice-Based Research Network
SICG: Serious iIllness Conversations Guide
SICP: Serious iIllness Conversations Program
